# Medical Ethics

**Published:** 1908-01

**Authors:** 


### MEDICAL ETHICS.

Pinard quotes Voltaire, that there is only one morality, as there is only one geometry. Very few people know geometry, but as soon as they study it a little they are all in accord. He protests against the remark he so frequently hears from patients: "I consulted a little doctor around the corner," or "a little country doctor where I was staying." He drew the picture of these "little doctors" forever traveling around the city streets and climbing staircases or jogging along the country roads, going, going all the time, never refusing their services, going into the wretched retreats of poverty, knowing well from dire experience that they can not rely upon being paid for their services, not even with gratitude, but going still, simply doing their duty as they see it. "These," Pinard remarks, "to my mind are the great physicians." Among other phases of medical ethics he discusses the question of responsibility, observing that few professions require such prompt decisions as the medical profession or impose such a burden of responsibility. He has known many physicians who gave up the idea of practicing after graduating with high honors, crushed by the weight of the responsibilities of a medical practice. Commenting on the fact that success does not always crown effort, even the most skillful, the most conscientious, he says that the physician is neither infallible nor all-powerful. In case of failure he must always expect to be blamed. In case of failure he may be arrested, treated as a malefactor and imprisoned, but the fear of blame should not deter him, even if it bring him in conflict with the courts of justice, if he acts according to the rules of the art. The question as to whether or not a physician should ever interrupt the course of a pregnancy, Pinard answers by saying that it is justifiable only when the mother is on the point of death from some affection caused or aggravated by the pregnancy. If the physician does not interfere, both mother and child will die, as the child is as fatally condemned as the mother. By interfering, the physician has a chance of saving the mother. Referring to the question of professional secrecy, Pinard says that it is a good symbol of the role of the physician in society, his task being to defend at the same time the interests of the individual and the interests of the public at large. Brouardel advised physicians to maintain the strictest professional secrecy at all times, but in daily life he frequently violated this rule, and Pinard thinks that there are many occasions when the physician would be acting contrary to his sense of right if he adhered strictly to the letter of the law. In case one of his clients, in a contagious stage of venereal disease, announced his approaching marriage, and could not be induced to postpone it, Pinard says that he would go directly to the father of the prospective bride and say to him: "Do not give your daughter in marriage to that young man." This is all he would say, but this would surely be enough. He might be sued for damages by his client, and he might be condemned by the courts, but his conscience would be at peace. He thinks that this mode of conduct should be taught in the medical schools. In this point he differs from other French writers on medical ethics. He believes that the time is coming when the matter of health on both sides of the marriage contract will be regarded more than at present, and that the family physicians of the two families will confer, as the lawyers now confer over the marriage settlement. On the other hand he declares that he would never denounce any one to the courts. He might testify that a man was the victim of poisoning, but he would never suggest who had done the crime. He would violate professional secrecy by reporting that a child to whom he had been called was the victim of inhuman treatment, and he would report to the authorities a case of epidemic disease, but no power could compel him to denounce the author of the crime. He would also warn a wet nurse that she would be incurring great risks if she took charge of a child whom he knew to have inherited syphilis, if the parents insisted on engaging her, against his warning. In regard to the question of fees, he says that the physician frequently gives advice to individuals and to communities which conflicts directly with his material interests. Members of no other profession do this, and the physicians thus occupy a place apart. In charging for his services, the physician should bear in mind the situation of his client and the importance of service rendered. The patient sometimes fails to recognize the importance of the service rendered, as when the physician discovers some affection and refers him to a surgeon, but the sharing of the fee is only justifiable, proper and ethical when it is done openly before the patient.—A. Pinard in Presse Medicale.

